# Temperature-Dependence of Lipid A Acyl Structure in *Psychrobacter cryohalolentis* and Arctic Isolates of *Colwellia hornerae* and *Colwellia piezophila*

**DOI:** 10.3390/md13084701

**Published:** 2015-07-30

**Authors:** Charles R. Sweet, Rebecca E. Watson, Corinne A. Landis, Joseph P. Smith

**Affiliations:** 1 Chemistry Department, 572M Holloway Road, United States Naval Academy, Annapolis, MD 21402, USA; E-Mails: rebeliz.wat@gmail.com (R.E.W.); clandis@hmc.psu.edu (C.A.L.); 2 Oceanography Department, 572C Holloway Road, United States Naval Academy, Annapolis, MD 21402, USA; E-Mail: jpsmith@usna.edu

**Keywords:** lipopolysaccharide, lipid A, lipid structure, mass spectrometry, psychrophile, homeoviscous adaptation

## Abstract

Lipid A is a fundamental Gram-negative outer membrane component and the essential element of lipopolysaccharide (endotoxin), a potent immunostimulatory molecule. This work describes the metabolic adaptation of the lipid A acyl structure by *Psychrobacter cryohalolentis* at various temperatures in its facultative psychrophilic growth range, as characterized by MALDI-TOF MS and FAME GC-MS. It also presents the first elucidation of lipid A structure from the *Colwellia* genus, describing lipid A from strains of *Colwellia hornerae* and *Colwellia piezophila*, which were isolated as primary cultures from Arctic fast sea ice and identified by 16S rDNA sequencing. The *Colwellia* strains are obligate psychrophiles, with a growth range restricted to 15 °C or less. As such, these organisms have less need for fluidity adaptation in the acyl moiety of the outer membrane, and they do not display alterations in lipid A based on growth temperature. Both *Psychrobacter* and *Colwellia* make use of extensive single-methylene variation in the size of their lipid A molecules. Such single-carbon variations in acyl size were thought to be restricted to psychrotolerant (facultative) species, but its presence in these *Colwellia* species shows that odd-chain acyl units and a single-carbon variation in lipid A structure are present in obligate psychrophiles, as well.

## 1. Introduction

The outer surface of the outer membrane of Gram-negative bacteria consists largely of lipid A, the required component and membrane anchor of endotoxin (lipopolysaccharide) [1]. This bioactive molecule is a major stimulus of the innate immune system during Gram-negative infection and septic shock, and as such, understanding of its structure is important, not just as an essential membrane lipid, but also in medical microbiology [2]. While the canonical *Escherichia coli* structure of lipid A is a potent pro-inflammatory agonist, other species of bacteria produce lipid A molecules that are weak agonists or even antagonists of the human immune system, with potential roles as therapeutics or vaccine adjuvants [3]. Constitutive evolutionary adaptation of endotoxin structure to the pathogenic, metabolic and environmental needs of the bacterium has been demonstrated in a variety of ways [4,5,6,7], and homeoviscous fluidity adaptation of membrane lipids, including lipid A, is a well-known response to temperature variation across a wide variety of organisms, including mesophilic bacteria. This metabolic phenomenon adapts the length and/or level of unsaturation present in membrane lipid acyl units, altering their melting point and, thus, the fluidity of the membrane. This protects the membrane from temperature-induced phase transitions that would disrupt the bilayer, compromising the structural integrity of the cell [8,9]. Adaptation of lipid fluidity in response to temperature has been characterized at the molecular level in *Bacillus*, *E. coli* and others [10,11,12], and much work has been done on desaturases, acyltransferases, transcription factors and other proteins involved in this response [13,14,15]. However, little is known about the role of metabolic temperature adaptation of lipid A structure in constitutively cold-adapted organisms, such as psychrophilic bacteria [16]. Previous work on the lipid A structure of psychrophiles has suggested that fluidity adaptation in the lipid A acyl moiety is primarily a constitutive evolutionary response [17] in which psychrophilic lipid A acylation does not change in a significant or consistent way with temperature [18]. The work presented here supports this assertion in obligate psychrophiles, organisms with growth temperature restricted to a range of 15 °C or lower. In contrast, however, we observe metabolic adaptation of lipid A in the facultative psychrophilic (psychrotolerant) organism *Psychrobacter cryohalolentis* in response to changes in growth temperature. This organism can grow in a range from −10 °C to at least 30 °C [19], and it is reasonable to hypothesize that purely constitutive adaptation of lipid A structure is insufficient to manage the fluidity of lipid A over such a large growth range. Consistent with this hypothesis, we determined that the acyl structure of lipid A shows a trend in structural alteration with growth temperature, including a general shortening in total length as well as decreased odd-chain acyl incorporation as the growth temperature of *P. cryohalolentis* is lowered. The structures proposed in this work are based on the isolation, purification and characterization of lipid A by negative-ion and positive-ion modes of matrix-assisted laser desorption/ionization time-of-flight (MALDI-TOF) MS, as well as fatty acid methyl ester (FAME) GC-MS. A variety of psychrophilic species were investigated, including two genera in the Alteromonadales (*Psychromonas marina* [20] and two *Colwellia* species) and one from the Moraxellaceae (*Psychrobacter*) [19]. The *Colwellia* species used are strains of *C. hornerae* [21] and *C. piezophila* [22] that were isolated from an arctic ice core as part of this work and identified by 16S rDNA sequencing.

## 2. Results

### 2.1. Structural Characterization of P. cryohalolentis Lipid A at 4 °C, 15 °C and 25 °C

**Figure 1:**
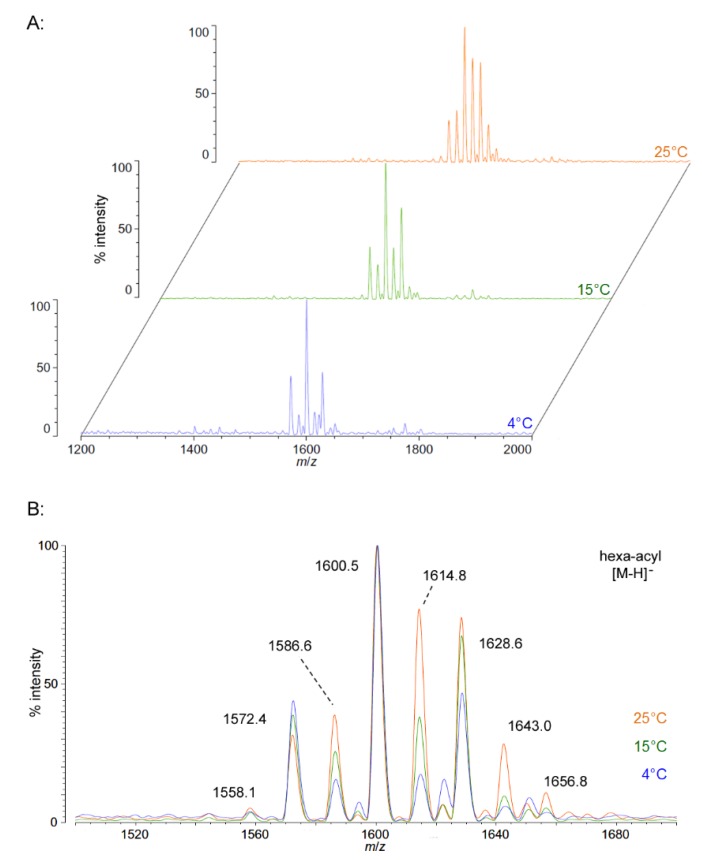
Negative ion MALDI-TOF MS of *P. cryohalolentis* lipid A at 4 °C, 15 °C and 25 °C. Lipid A was prepared as described in the Materials and Methods. Spectral data were collected by Shimadzu Axima Confidence MS with a power setting of 75–80 and pulsed extraction at 2000 Da as the average of at least 1000 profiles in the linear mode, displayed in 3D offset comparison by temperature (**A**) or as a stack of the main peak region (**B**). Peak interpretation is shown on the spectra and in Table 1.

We isolated *P. cryohalolentis* lipid A from cultures grown at 4 °C, 15 °C or 25 °C by the modified Bligh-Dyer method [23] and characterized the structure of this lipid by MALDI-TOF MS and FAME GC-MS. *P. cryohalolentis* produces a surprisingly diverse set of lipid A acyl forms at 25 °C, as has been previously reported [17]. Negative ion MALDI-TOF MS of this lipid A reveals at least six significant acyl variants differing by single methylenes (CH_2_) in the dominant penta-acyl set of structures, centered on the most abundant [M − H]^−^ peak at an *m*/*z* ratio of 1600.5 (Figure 1), corresponding to the known molecular mass of the predominant *P. cryohalolentis* lipid A of 1601.99 Da (Table 1). The well-characterized lipid A from *E. coli* W3110 was used as a control and a standard for all procedures in this work, and structural examples of the negative and positive ions characteristic of lipid A in MALDI-TOF MS, as well as a summary of lipid A nomenclature can be found in the *E. coli* control spectra (Figure S1).

As growth temperature decreases, *P. cryohalolentis* lipid A exhibits consistent alterations in its suite of acyl length variants. Negative ion MALDI-TOF MS at 15 °C shows a significantly lower proportion of the forms containing odd-length acyl chains (the peaks at *m*/*z* 1586.6 and 1614.8), less of the peak that is +2(CH_2_) larger than average (*m*/*z* 1628.6), more of the peak −2(CH_2_) smaller than average (*m*/*z* 1572.4) and almost complete loss of the peaks +3(CH_2_) and +4(CH_2_) larger than average (*m*/*z* 1643.0 and 1656.8), while the minor peak −3(CH_2_) lesser than average (*m*/*z* 1558.1) is not reduced with temperature. Negative ion MALDI-TOF at 4 °C reveals that these shifts show a trend in proportion with temperature; the changes described above at 15 °C are continued and extended in the structure of lipid A from organisms grown at 4 °C, and comparison of the acyl distribution patterns of the highest and lowest temperatures indicates a significant shift to smaller acyl forms as the growth temperature drops. As an illustration of this, Figure 1B shows that the +1(CH_2_) and +2(CH_2_) flanking peaks, which are nearly as large as the main peak at 25 °C, are reduced markedly at 4 °C to the point that they are equal to the amounts of the −1(CH_2_) and −2(CH_2_) peaks) and also shows the nearly complete loss of odd-length acyl chain incorporation into lipid A at cold temperatures.

**Table 1:** Summary of MALDI-TOF MS observations and predicted masses of ions.

Molecule	[M − H]^−^ Predicted Mass (Da)	[M − H]^−^ Observed *m*/*z*	B1^+^ Predicted Mass (Da)	B1^+^ Observed *m*/*z*	B2^+^ Predicted Mass (Da)	B2^+^ Observed *m*/*z*	[M + Na]^+^ Predicted Mass (Da)	[M + Na]^+^ Observed *m*/*z*
***E. coli* hexa-acyl**	**1797.36**	**1797.5**	**1087.51**	**1087.6**	**1701.38**	**1701.1**	**1821.35**	**1821.57**
***P. cryohalolentis*** −(CH_2_)_3_	1558.91	1558.1	905.17	--	1462.93	--	1582.90	1582.4
−(CH_2_)_2_	1572.93	1572.4	919.19	919.1	1476.95	1476.4	1596.93	1596.6
−CH_2_	1586.96	1586.6	933.22	933.3	1490.98	1490.5	1610.96	1610.8
**hexa-acyl**	**1600.98**	**1600.5**	**947.25**	**947.2**	**1505.01**	**1504.5**	**1624.98**	**1624.7**
+CH_2_	1615.01	1614.8	961.27	961.4	1519.03	1518.4	1639.01	1639.5
+(CH_2_)_2_	1629.04	1628.6	975.30	975.3	1533.06	1532.6	1653.04	1652.9
+(CH_2_)_3_	1643.06	1643.0	989.33	--	1547.09	--	1667.06	1667.7
***C. piezophila*** −(CH_2_)_3_	1428.76	1429.5	777.00	777.7	1332.78	1334.3	1452.76	1454.8
−(CH_2_)_2_	1442.79	1443.0	791.02	791.7	1346.81	1348.1	1466.79	1468.4
−CH_2_	1456.82	1456.3	805.05	805.7	1360.84	1361.5	1480.81	1481.4
**penta-acyl**	**1470.84**	**1470.0**	**819.08**	**819.7**	**1374.86**	**1374.6**	**1494.84**	**1495.1**
+CH_2_	1484.87	1483.9	833.10	833.3	1388.89	1388.4	1508.87	1509.1
+(CH_2_)_2_	1498.90	1497.5	847.13	847.0	1402.92	1402.2	1522.89	1522.9
+(CH_2_)_3_	1512.92	1511.1	861.15	--	1416.94	*	1536.92	1536.9
***C. hornerae***−(CH_2_)_3_	1400.71	1401.5	748.94	--	1304.73	1306.4	1424.71	1426.7
−(CH_2_)_2_	1414.74	1414.4	762.97	763.3	1318.76	1319.3	1438.73	1439.7
−CH_2_	1428.76	1428.3	777.00	777.4	1332.78	1333.2	1452.76	1453.4
**penta-acyl**	**1442.79**	**1442.1**	**791.02**	**791.0**	**1346.81**	**1346.2**	**1466.79**	**1467.0**
+CH_2_	1456.82	1456.1	805.05	805.5	1360.84	*	1480.81	1481.3
+(CH_2_)_2_	1470.84	1469.9	819.08	816.3	1374.86	*	1494.84	14495.5
+(CH_2_)_3_	1484.87	1483.8	833.10	--	1388.89	*	1508.87	1509.7

Calibrated mass/charge ratios (observed mass) of major ions in the MALDI-TOF MS spectra are compared to the calculated molecular weight (predicted mass) of each ion based on the proposed lipid A structures; “+” and “−” in the molecule description column indicate observed variation in the size of the molecular ion, not fragmentation/ionization; Characteristic fragmentation/ionization identities are described in each of the data column headers; -- = not detected; bold masses indicate the most abundant species in each structure; * If present, these peaks are buried in noise and/or the tail of the [M + Na]^+^ acyl variants.

**Figure 2:**
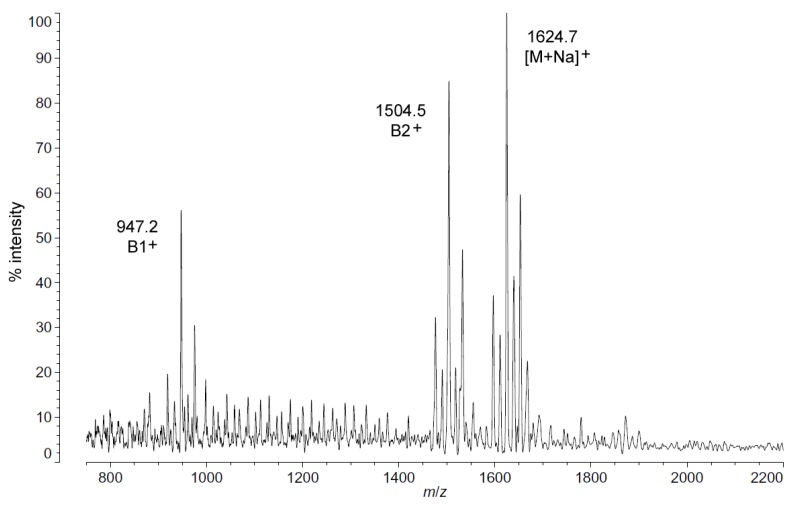
Positive ion MALDI-TOF mass spectra of *P. cryohalolentis* lipid A at 4 °C. Lipid A was prepared as described in the Materials and Methods. Spectral data were collected by Shimadzu Axima Confidence MS with a power setting of 80, pulsed extraction and 1500 profiles in the linear mode. Peak interpretation is shown on the spectra and in Table 1.

Positive ion MALDI-TOF MS (Figure 2) also shows this shift in pattern at low temperatures in the structure of the characteristic B1^+^, B2^+^ and [M + Na]^+^ ions. These fragments and adducts are centered on *m*/*z* of 947.2, 1504.5 and 1624.7, respectively. They correspond to the predominant molecular mass of 1601.99 Da (Table 1) and aid in establishing the acyl distribution of *P. cryohalolentis*, as has been previously shown [17]. The altered acyl pattern shown in Figure 1 as characteristic of cold-grown *P. cryohalolentis* is clear as well in the B1^+^ and B2^+^ ions of Figure 2, though it is a bit obscured in the [M + Na]^+^ cluster due to the presence of [M + K]^+^ adducts (the difference of ~16 Da between sodium and potassium overlaps with that of the ~14 Da difference between acyl variants). Figure S2 shows the positive ion spectrum at 25 °C for comparison, in which the large amounts of the +1(CH_2_) and +2(CH_2_) flanking peaks characteristic of room-temperature *P. cryohalolentis* growth are evident.

FAME GC-MS results are consistent with the acyl structural changes in *P. cryohalolentis* at the cold temperatures that are described above. At 25 °C, the lipid A-derived FAMEs seen in *P. cryohalolentis* include odd-chain 3-OH undecanoyl methyl ester, 3-OH tridecanoyl methyl ester, tridecanoyl methyl ester and pentadecanoyl methyl ester, as well as even-chain FAMEs (Table 2), while at 4 °C, only the pentadecanoyl methyl ester and the even-chain acyl units remain.

### 2.2. Structural Characterization of C. piezophila Lipid A at 4 °C and 15 °C

**Figure 3:**
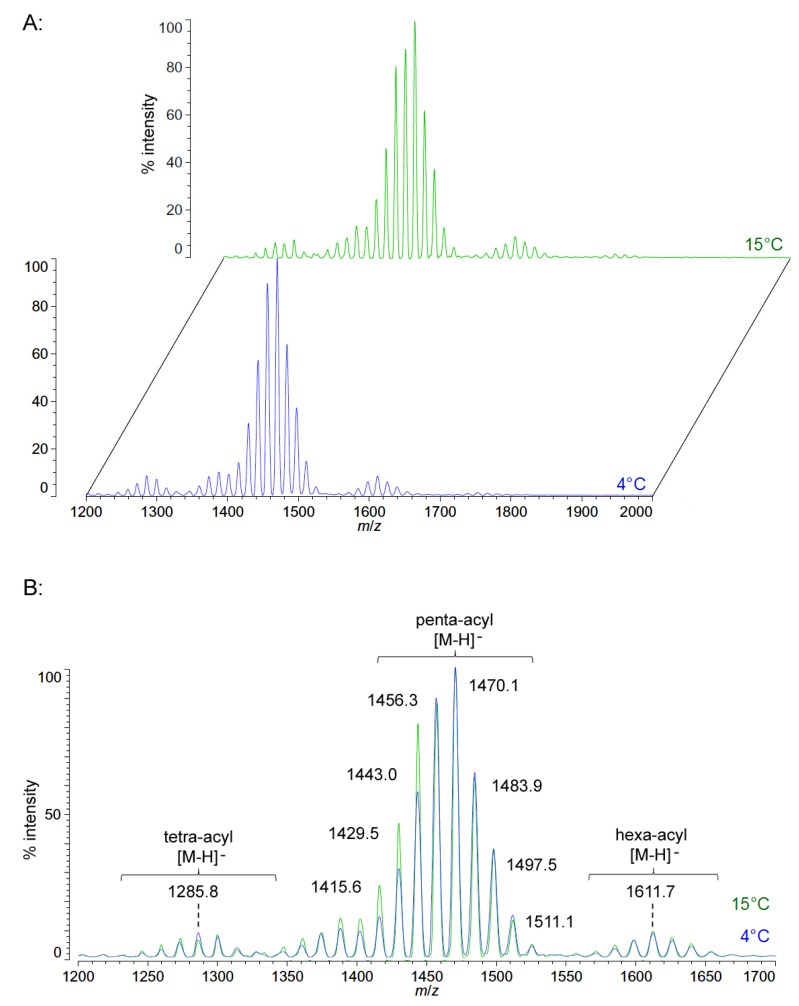
Negative ion MALDI-TOF mass spectra of *C. piezophila* lipid A at 4 °C and 15 °C. Lipid A was prepared as described in the Materials and Methods. Spectral data were collected by Shimadzu Axima Confidence MS with a power setting of 75 and pulsed extraction at 2000 Da as the average of at least 1000 profiles in the linear mode in offset comparison by temperature (**A**) and as a stack of the main peak region (**B**). Peak interpretation is shown on the spectra and in Table 1.

**Figure 4:**
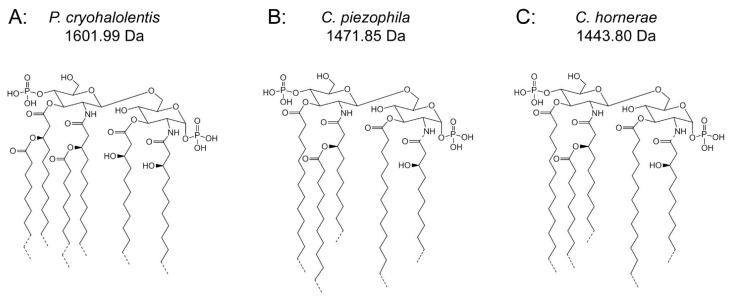
Proposed psychrophilic lipid A structures. These represent the most likely average structures of the most abundant molecular forms of lipid A in *P. cryohalolentis* (**A**), *C. piezophila* (**B**) and *C. hornerae* (**C**) based on the summation of all structural data. All of these organisms show significant variability in acyl chain length; hashed bonds indicate non-stoichiometric substituents that give rise to the major molecular forms.

Unlike *P. cryohalolentis* lipid A, the obligate psychrophile *C. piezophila* does not show any alteration of lipid A acyl size or distribution with respect to temperature. Negative ion MALDI-TOF MS of *C. piezophila* lipid A at both 4 °C and its upper growth limit of 15 °C show essentially identical spectra. However, this species’ lipid A is unusual in that it has an even greater variety of single-methylene acyl length variant forms than *P. cryohalolentis.* The most abundant negative ion [M − H]^−^ peak at *m*/*z* 1470.1 is flanked by many other significant forms, all differing by ~14 mu, indicative of single methylene differences in chain length of the acyl units (Figure 3 and Table 1). There are also less abundant groups of single-methylene variants clustered around main peaks with alternative numbers of acyl chains, including a tetra-acyl cluster centered on *m*/*z* 1285.8 and a hexa-acyl one centered on *m*/*z* 1611.7. The mass gained (+Δ141.7 mu) or lost (−Δ184.3 mu) between the most abundant peak of the main cluster (a predicted molecular mass of 1471.85 Da) and those of these lesser clusters is useful in determining the details of the chemical structure of this lipid A, as described in the Discussion Section and shown in Figure 4.

**Table 2:** FAME GC-MS observations and structural interpretations.

Acyl Residue	Standard Retention Time	*E. coli*	*P. cryohalolentis*		*C. piezophila*	*C. hornerae*	
			*at 4 °C*	*at 25 °C*		
*Lipid A-derived 3-hydroxylated fatty acids:*						
3-hydroxydecanoate (3-OH C10:0)	3.7 *	--	--	--	3.680	3.680
3-hydroxyundecanoate (3-OH C11:0)	4.2 *	--	--	4.220	4.220	4.250
3-hydroxydodecanoate (3-OH C12:0)	4.717	--	4.727	4.727	4.727	4.727
3-hydroxytridecanoate (3-OH C13:0)	5.2 *	--	--	5.187	5.187	5.191
3-hydroxytetradecanoate (3-OH C14:0)	5.600	5.600	--	--	--	--
*Lipid A-derived non-hydroxylated fatty acids:*						
decanoate (C10:0)	2.887	--	--	2.907	2.912	--
dodecanoate (C12:0)	3.979	3.973	3.987	3.987	3.980	--
tridecanoate (C13:0)	4.493	--	--	4.500	--	--
tetradecanoate (C14:0)	4.971	4.967	4.967	4.973	4.970	4.973
pentadecanoate (C15:0)	5.400	--	5.400	5.400	5.400	5.400
*Phospholipid-derived fatty acids:*						
hexadecanoate (C16:0)	5.793	5.787	5.793	5.793	5.793	5.793
hexadecenoate (C16:1)	5.727	--	5.720	5.720	5.722	5.720
heptadecanoate (C17.0)	6.153	--	--	6.153	6.152	6.153
heptadecenoate (C17.1)	6.1 *	--	--	6.080	--	--
octadecanoate (C18:0)	6.493	6.487	6.493	6.493	6.493	6.493
octadecenoate (C18:1)	6.413	6.413	6.420	6.420	6.420	6.420
octadecadienoate (C18:2)	6.365	--	6.360	6.360	6.360	6.360	

Retention times of fatty acid methyl esters in comparison to authentic standards; Identities were determined by comparison to standards, EI-MS interpretation and library matching (see text); * Retention times for which standards are not available were predicted by interpolation of FAMEs included in bacterial fatty acid methyl ester (BAME) standard; -- = not detected.

**Figure 5:**
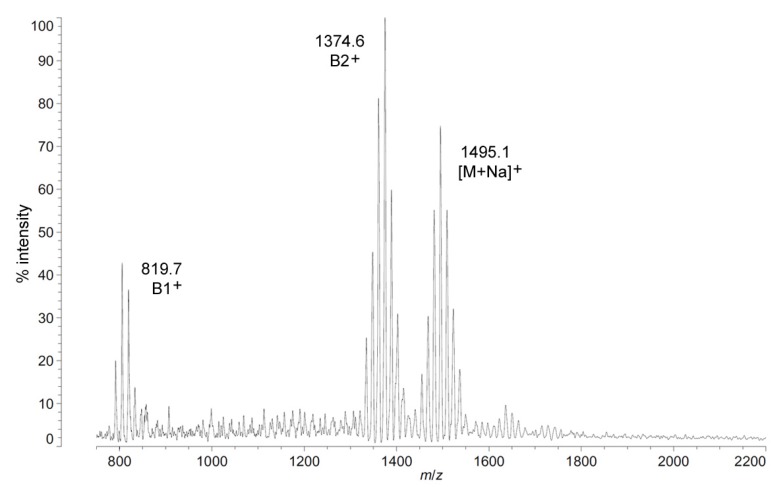
Positive ion MALDI-TOF mass spectra of *C. piezophila* lipid A at 4 °C. Lipid A was prepared as described in the Materials and Methods. Spectral data were collected by Shimadzu Axima Confidence MS with a power setting of 75, pulsed extraction, and 2000 profiles in the linear mode. Peak interpretation is shown on the spectra and in Table 1.

The positive ion MALDI-TOF MS of *C. piezophila* lipid A shows the same clustering pattern as the negative ion mode (Figure 5 and Table 1). As with the distances between tetra-, penta- and hexa-acyl variants in the negative ion mode, the differences between the B1^+^ and B2^+^ ions of *C. piezophila* inform about the locations and sizes of the acyl units. The B1^+^ ion cluster of this spectrum shows at least four single-methylene variants in acyl length, centered on a most abundant peak of *m*/*z* 819.7. This is 554.9 mu smaller than the most abundant ion in the B2^+^ ion cluster, at *m*/*z* 1374.6. This mass difference indicates that the glucosamine I fragment lost to generate the B1^+^ ion is a diacylglucosamine, and the remaining glucosamine II fragment must therefore be triacylated. The third cluster in the spectrum, centered on *m*/*z* 1495.1, represents the sodium adduct [M + Na]^+^ of the dominant penta-acyl molecular structure. B2^+^ and [M + Na]^+^ clusters are also present for the hexa-acyl form of *C. piezophila* lipid A; however, if the B1^+^ ion is distinct from that of the penta-acyl species (*i.e.*, if the sixth fatty acid is on the non-reducing glucosamine II end of the structure), it cannot be observed due to the high background of the lower region of the positive ion spectrum. Similarly, no positive ions can be discerned that would correspond to the tetra-acyl structure seen in the negative mode.

FAME GC-MS analysis of *C. piezophila* lipid A determined the acyl composition of this lipid A and displays some differences from the other bacterial species in this study that are critical to understanding the proposed structure shown in Figure 4B. Most distinctively, the set of hydroxylated FAMEs seen in *C. piezophila* are shorter even than those seen in *P. cryohalolentis*. While *P. cryohalolentis* has only a small amount of 3-OH undecanoyl methyl ester, this length is the most abundant hydroxy-acyl chain in *C. piezophila*, along with lesser amounts of 3-OH decanoyl methyl ester and 3-OH dodecanoyl methyl ester and a trace of 3-OH tridecanoyl methyl ester.

Tetradecanoyl methyl ester dominates the lipid A-derived non-hydroxy acyl units, with lesser amounts of pentadecanoyl methyl ester, dodecanoyl methyl ester (but no tridecanoyl methyl ester) and decanoyl methyl ester observed, as well. Combined with the MALDI-TOF data, we therefore propose a plausible average molecular structure in Figure 4B with 3-OH undecanoyl and tetradecanoyl primary acyl chains and a dodecanoyl acyl-oxyacyl (secondary) unit.

### 2.3. Structural Characterization of C. hornerae Lipid A at 4 °C and 15 °C

**Figure 6:**
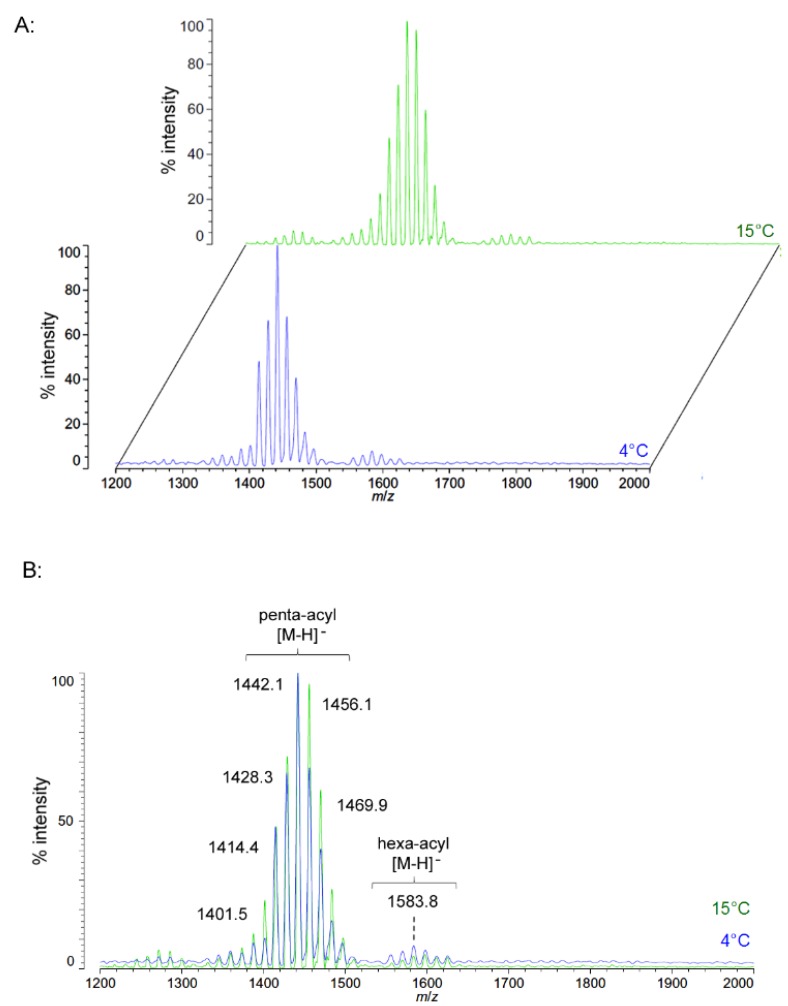
Negative ion MALDI-TOF mass spectra of *C. hornerae* lipid A at 4 °C and 15 °C. Lipid A was prepared as described in the Materials and Methods. Spectral data were collected by Shimadzu Axima Confidence MS with a power setting of 75–80 and pulsed extraction at 2000 Da as the average of at least 1000 profiles in the linear mode in offset comparison by temperature (**A**) and as a stack of the main peak region (**B**). Peak interpretation is shown on the spectra and in Table 1.

Negative ion MALDI-TOF spectra of lipid A from another obligate psychrophile, *C. hornerae*, also shows a very similar overall pattern and general range of acyl length variants to that of *C. piezophila* (Figure 6). There is a slight difference in that the most abundant [M − H]^−^ ion of *C. hornerae*, at *m*/*z* 1441.9, is ~28 mu smaller than that of *C. piezophila* and corresponds instead to a predicted molecular mass of 1442.79 Da. However, the general range of the *C. hornerae* variants seen in Figure 6 and the acyl content assessed by FAME GC-MS (Table 2) are similar to that of *C. piezophila.* This suggests that the difference in average size of lipid A between these species is due to minor alterations in enzymatic specificity for greater use of shorter acyl units, rather than a significant shift in the acyl content.

**Figure 7:**
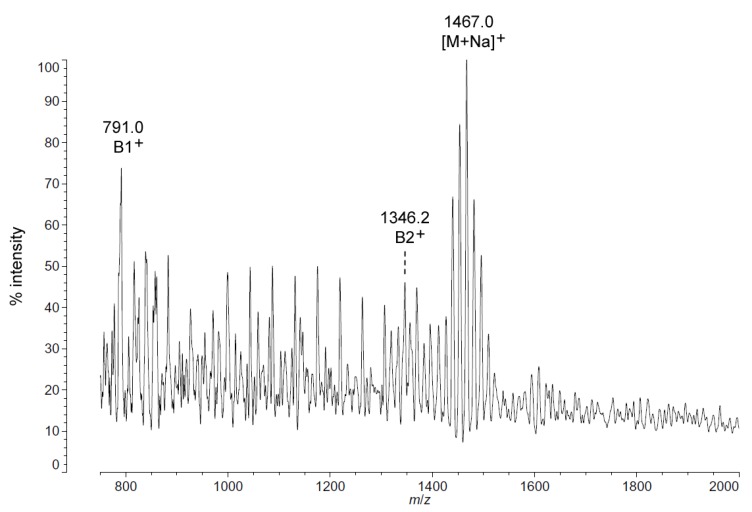
Positive ion MALDI-TOF mass spectra of *C. hornerae* lipid A at 4 °C. Lipid A was prepared as described in the Materials and Methods. Spectral data were collected by Shimadzu Axima Confidence MS with a power setting of 75, pulsed extraction, and 2000 profiles in the linear mode. Peak interpretation is shown on the spectra and in Table 1.

As with *C. piezophila*, the positive ion MALDI-TOF spectrum of lipid A from *C. hornerae* confirms the proposed molecular masses with those of the B2^+^ and [M + Na]^+^ clusters, centered on *m*/*z* of 1346.2 and 1467.0, respectively (Figure 7), and informs about the acyl distribution of the molecule through the mass difference of 555.2 mu between the B1^+^ and B2^+^ ion clusters (loss of a diacylglucosamine fragment). Furthermore, as with *C. piezophila*, the relative abundance of 3-OH undecanoyl methyl ester and tetradecanoyl methyl ester in the FAME GC-MS of *C. hornerae* suggests that these are the most abundant acyl units in the molecule on average (Figure 4C), with greater or lesser acyl units in combination to account for all of the acyl length variants seen in Figure 6.

### 2.4. Structural Characterization of P. marina Lipid A at 4 °C and 15 °C

Similar to *C. piezophila* and *C. hornerae*, lipid A from the previously-characterized obligate psychrophile *P. marina* [17] does not demonstrate any alteration of the acyl size or distribution of lipid A with temperature. Negative ion MALDI-TOF MS of *P. marina* lipid A at both 4 °C and its upper growth limit of 15 °C shows essentially identical spectra (Figure S3), with two main peaks of relatively equal distribution. The [M − H]^−^ ions of penta-acyl and hexa-acyl *P. marina* lipid A appear at *m*/*z* 1610.3 and 1792.8, respectively, corresponding to the previously-reported lipid A molecular masses of 1611.02 Da and 1793.33 Da. The positive ion MALDI-TOF MS spectrum confirms these molecular masses and also establishes the acyl distribution of *P. marina* lipid A, again in confirmation of the previously-reported structure (Figure S4).

### 2.5. Discussion

We find that the facultative (Psychrotolerant) species *P. cryohalolentis* synthesizes lipid A with a dominant hexa-acyl cluster with at least six significant lipid A structures varying from each other by single methylene (CH_2_) units at 25 °C and minor clusters of the penta-acyl and hepta-acyl forms (Figure 1; and also [17]). These variations are generated by use of a wide range of acyl chain lengths, including C11:0–C13:0 3-OH acyl units in primary linkage and C10:0–C15:0 in secondary linkage. The structural form represented in Figure 4A shows the most likely acyl lengths, on average, which would generate the most abundant structure (1601.99 Da), though a diversity of discrete acylation forms is likely to populate each of the observed molecular weights. In contrast to the obligate psychrophiles characterized in this work, *P. cryohalolentis* lipid A displays modulation of the acyl elements of lipid A structure with varying growth temperature. Presumably, these changes are the result of a combination of temperature-dependent changes in the activity or transcription of enzymes synthesizing fatty acids and/or lipid A, as has been noted in the fluidity adaptation of other bacterial species in response to temperature changes [13,14].

The *P. cryohalolentis* K5 genome (http://www.ncbi.nlm.nih.gov) encodes one homolog of the lipid A acyltransferases LpxD, LpxL and LpxM, while containing two homologs of LpxA, suggesting that the 3 and 3′ primary acyl positions of the molecule might be particularly subject to variability in acyl usage as temperature changes. This is analogous to the switch from LpxL to LpxP activity in *E. coli* at low temperature, which is responsible for replacing dodecanoyl (C12:0) acyl units with hexadecenoyl (C16:1) ones during cold shock [10,14]. These changes to overall lipid A structure with descending temperature, while subtle enough that 1601.99 Da is still the predominant molecular weight at all temperatures, have distinct and significant effects on many of the flanking acyl variants of *P. cryohalolenti*s lipid A (Figure 1). The first of these effects is the near-elimination of odd-length acyl chains in lipid A and in phospholipids at cold temperature (seen in both the absence of single-methylene flankers of the most abundant peak in Figure 1 and Figure 2 and also in the absence of most of the odd-chain residues from the FAME GC-MS results in Table 2), and the second is a significant shift toward a shorter overall average of the acyl units at cold temperature, which demonstrates a structural trend in fluidity adaptation of psychrophilic lipid A with temperature. While the metabolic benefit of changes to the odd-chain population of acyl units in *P. cryohalolentis* with temperature is unclear, the appearance of this effect in the acyl residues of both lipid A and phospholipids suggests that this is happening at the level of fatty acid synthesis. The trend in overall shortening of the lipid A acyl units seen in the MALDI-TOF data is, however, a homeoviscous adaptation of the lipid A acyl moiety in response to the challenge to membrane fluidity posed by cold growth temperatures, similar to well-characterized shifts in the acyl fluidity of membrane phospholipids in psychrophiles and other organisms [8,9,24]. While it is tempting to speculate that the existence of two LpxA homologs in *P. cryohalolentis* implies a molecular mechanism for the strong change in odd-chain acyl incorporation with temperature, the substrate specificities of these homologs remains to be investigated and will be an interesting aspect of future work in psychrophilic lipid A. It will also be fruitful to see if ultra-cold growth of these psychrophiles (at −5 to −10 °C) alters *P. cryohalolentis* further or alters *Colwellia* or *P. marina* lipid A. However, growth at such temperatures requires specialized equipment beyond the capacity of the current investigation.

Figure 4B,C demonstrates the most likely average acyl length and distribution of lipid A from *C. piezophila* and *C. hornerae*, two organisms cultured from the sea ice-water interface of Arctic fast ice [25]. Both of these organisms are obligate psychrophiles (they cannot grow above 15 °C). In addition, the *C. piezophila* strain BRX10.3 described here is remarkable in that while it is psychrophilic, it grows at nominal atmospheric pressure and up to 15 °C, both of which are traits that distinguish it from the type strain of the *C. piezophila* species, which is an obligate barophile [22]. Both of these *Colwellia* strains demonstrate an astonishing diversity of lipid A acyl forms, with a main penta-acyl cluster of at least eight significant forms (and perhaps as many as fourteen, including minor ones), in addition to lesser clusters of at least five tetra-acyl and five hexa-acyl variants (Figure 3). In *C. piezophila*, the most abundant molecular structure is a penta-acyl variant with a mass of 1471.85 Da. Unexpectedly, this mass (and its characteristic positive ion fragments seen in Figure 5) can only be reasonably explained in light of known lipid A biosynthetic pathways by inclusion of normal (non-hydroxylated) fatty acids in primary linkage to the carbohydrates, a variation previously only known to occur in the presence of tetradecanoyl residues at the 3 and 3′ position of *Chlamydia trachomatis* lipid A [26,27], due to the unusual acyl specificity of *C. trachomatis* LpxA [23]. FAME GC-MS shows that tetradecanoyl residues are also the most abundant non-hydroxylated acyl unit in *C. piezophila* lipid A; therefore, this chain length is shown in primary linkage in the proposed structure in Figure 4B. In this structure, the non-hydroxylated residues are placed at the 3 and 3′ positions in light of the known positioning of this acyl unit in *C. trachomatis*. Alternative structural explanations of *C. piezophila* lipid A that would place the non-hydroxylated acyl units solely in their typical acyl-oxyacyl positions can be ruled out by the fact that a mass of 1471.85 Da requires two 3-OH-acyl units and three non-hydroxylated acyl units, a biosynthetic impossibility if none of the primary linkages are non-hydroxylated chains. Furthermore, the dominant B1^+^ ion mass of *m*/*z* 820.1 (Figure 5 and Table 1) shows that two non-hydroxylated units (and one hydroxy-acyl unit) are present on the non-reducing (glucosamine II) end of the molecule.

The minor hexa-acyl components of *C. piezophila* lipid A represent the addition of another non-hydroxylated acyl unit with an average gain in total acyl length of nine carbons for the most abundant peak seen at *m*/*z* 1611.7 (Figure 3). However, it is unlikely that this is the result of the simple addition of a nonanoyl unit to the penta-acyl structure. This hexa-acyl cluster likely involves some biasing of the size of other acyl residues toward shorter lengths, particularly substitution of tetradecanoyl residues for dodecanoyl and/or tridecanoyl ones, which, unlike nonanoyl residues, are present in the FAME GC-MS analysis of *C. piezophila* (Table 2). To complete the most likely average structure corresponding to the most abundant molecular form at 1470.84 Da shown in Figure 4B, a fifth acyl unit of dodecanoyl length is placed in acyl-oxyacyl linkage on glucosamine II, consistent with the spectral evidence described above. In light of the presence of an LpxL/P homolog rather than an LpxM homolog in the *C. piezophila* genome (http://www.ncbi.nlm.nih.gov), this secondary acyl residue is placed at the *N*-linked (2′) position of the structure. The final variation in *C. piezophila* lipid A structure, the loss of an acyl unit to yield the minor tetra-acyl cluster seen at *m*/*z* of 1285.8, is explained by the removal of one of the hydroxy-acyl units in primary linkage. On average, the mass difference is that of a 3-OH undecanoyl residue, which is the most abundant hydroxy-acyl residue seen in the *C. piezophila* FAME GC-MS analysis (Table 2). This alteration is likely to be the result of a lipid A deacylase similar in activity to PagL or LpxO from other Gram-negative species [28,29]; however, no homolog of the *O*-acyl specific PagL or LpxO deacylases is present in the *C. piezophila* genome. This suggests that the hydroxy-acyl unit removed in this process is on the amide-linked position of the reducing sugar (indicated in Figure 4 with a hashed bond rather than a solid one) and that the ability to synthesize the tetra-acyl structure of *Colwellia* is due to an uncharacterized 2-*N*-acyl lipid A deacylase.

Analysis of *C. hornerae* lipid A (Figure 4C) is identical in almost all respects to that of *C. piezophila*, with the exception that the average size of *C. hornerae* is ~28 mu (two methylene units) smaller than the corresponding structures in *C. piezophila*, suggesting a shift in the substrate specificity of one or more of the *C. hornerae* acyltransferases in comparison to those of *C. piezophila*(see Figure 5 and Figure 6). Comparison of *C. piezophila* and *C. hornerae* to related mesophilic gamma-proteobacteria, such as *E. coli* (Figure S1) or *Vibrio* species [30,31], shows that the *Colwellia* lipid A contains generally shorter acyl units, much like *P. cryohalolentis* in comparison to its close relative, *Acinetobacter baumannii* [17].

These comparisons suggest that in the case of obligate psychrophiles, evolutionary adaptation to cold environments has constitutively altered the structure of lipid A in these organisms to use more fluid acyl chains than their mesophilic relatives, rather than relying on metabolic responses to tune the fluidity of the outer membrane to temperature. Facultative psychrotolerant organisms, such as *P. cryohalolentis*, conversely, require greater capability to respond to and thrive in a wider range of temperatures. The ability of this species to metabolically alter the type and length of its lipid A acyl units is likely to be an aspect of this greater adaptability, increasing the functional temperature range of the outer membrane in these organisms. Thus, psychrotolerant species, if not the obligate psychrophiles, join the ranks of those organisms capable of metabolic changes to the fluidity of lipid A acyl moiety of the outer membrane.

## 3. Materials and Methods

### 3.1. Culture Conditions, Strains and Reagents

*E. coli* W3110 was grown at 37 °C in lysogeny broth (Fisher brand LB-Miller) with 215-rpm rotatory shaking for 24 h as a control strain for all experiments. *P. marina* (ATCC BAA-724) and primary culture strains *C. hornerae* BRX8.1 and *C. piezophila* BRX10.3 were grown in Difco Marine 2216 medium at 4 °C and 15 °C with 215-rpm rotatory shaking for 24–72 h until stationary phase growth yield was obtained. Cultures were pre-conditioned to each temperature by growth on Marine 2216 medium agar plates for at least several days prior to liquid growth. *P. cryohalolentis* K5 (ATCC BAA-1226) was grown in marine medium at 4 °C, 15 °C and 25 °C with 215-rpm rotatory shaking for 24–96 h until stationary phase growth yield was obtained. All organisms were grown at varied temperatures from a low growth temperature of 4 °C to the maximum incubator temperature tolerated by the strain, 15 °C for *P. marina* and *Colwellia* spp., 25 °C for *P. cryohalolentis* (though some have reported higher temperature tolerance for *P. cryohalolentis*, strain BAA-1226 did not grow at 37 °C and grew on plates, but not in liquid culture at 32 °C in these experiments). All media were purchased as dehydrated mixtures; culture plates were prepared by mixing media with 1.5% w/v Difco agar. Bulk solvents, reagents and standards were purchased from Fisher Scientific International, Inc. (Pittsburgh, PA, USA) or Sigma-Aldrich (St. Louis, MO, USA) and were HPLC-grade or better. Bioinformatics of Lpx biosynthetic genes in *P. cryohalolentis* and *Colwellia* genomes were assessed using NCBI’s microbial genomes databases and BLAST search algorithms (http://www.ncbi.nlm.nih.gov).

### 3.2. Isolation of the Arctic Strain of Colwellia hornerae BRX8.1 and Colwellia piezophila BRX10.3

Cores of shore-fast sea ice were taken from the Chukchi Sea in the vicinity of Barrow AK on 12 March 2012 as part of the BROMEX 2012 field campaign [25]. These cores were processed by melting successive sections into 150-mL petri dishes in a sterile hood and collecting the meltwater in sterile 50-mL tubes. These tubes were stored and transported on blue ice (0 °C) to the United States Naval Academy (USNA), where ~100-µL samples were spread on Marine 2216 medium agar plates and incubated at 15 °C. Colonies were purified several times by single-colony isolation and then stored as glycerol cryo-stocks for later 16S RNA identification and lipid isolation. A total of 19 psychrophilic *Colwellia* strains were isolated from the bottom 10-inch section of the core, which was the seawater/ice interface. None were isolated from other sections of the core.

### 3.3. Gene Sequencing and Identification of Arctic Colwellia Strains

Commercial 16S RNA gene sequencing (GENEWIZ, South Plainfield, NJ, USA) was performed to identify purified bacterial strains cultured from the BROMEX biosampling. Most of these strains were identified to be species of *Colwellia*, including the strains characterized in this work. The 16S RNA sequences for these strains (Figure S5) were 100% identity matches by the BLAST algorithm (using the NCBI “nr/nt” curated nucleotide database, http://www.ncbi.nlm.nih.gov) to the 16S sequence of *C. hornerae* (BRX8.1) and *C. piezophila* (BRX10.3). BRX10.3 is unique among the BROMEX cultures, with only 97% 16S sequence identity to the next closest isolate. However, this strain of *C. piezophila* does not exhibit obligate barophilic growth and can grow up to 15 °C, unlike the type strain [22], demonstrating that it is a novel strain variant of *C. piezophila*. All other Gram-negative isolates were characterized to be strains of *C. hornerae* and are identical, or nearly so, to BRX8.1 in the 16S sequence. 16S rDNA sequences of BRX8.1 and BRX10.3 have been deposited with GenBank Accession Numbers KR349200 and KR349201, respectively.

### 3.4. Lipid A Isolation and DE52 Purification

A bacterial cell mass of 100–250 mL (depending on species) of liquid shaking culture was collected by centrifugation at 2600× *g* and resuspended in phosphate-buffered saline. This suspension was mixed with chloroform and methanol to form single-phase solution (1:2:0.8 C/M/aq) as the starting point for two-phase Bligh–Dyer extraction with mild acid release of lipid A, conducted as described previously [23]. Crude lipid A isolate was purified by DE52 anion-exchange chromatography and by further Bligh–Dyer extraction, also as described previously [23].

### 3.5. MALDI-TOF MS

Purified lipid A samples were dissolved in a small amount (30–100 μL) of 2:1 chloroform/methanol. A sample of this solution was mixed *in situ* on a MALDI target slide with 20 mg/mL 6-aza-2-thiothymine in 9:9:2 water/acetonitrile/10% ammonium citrate in ratios ranging from 4:1 to 1:4, with a maximum total load volume of 2 μL. Spectral data were collected by Shimadzu Axima Confidence MALDI-TOF MS in the negative or positive linear mode (pulsed extraction 2000 Da) as the average of up to 2000 shots at moderate power (75–90 arbitrary units; see the individual figure legends for details) calibrated with standard mass controls (ProteoMass, Sigma-Aldrich, St. Louis, MO, USA). Shimadzu MALDI-MS software was used to perform the following processing: (1) fine mass calibration to mode-specific standard datasets built from spectral data for *E. coli* W3110 lipid A (per the manufacturer instructions); (2) averaging of all profiles collected for each sample; and (3) processing using manufacturer-recommended settings for display of averaged molecular weight peaks from data collected with isotopic resolution: baseline filter width of 100 channels, threshold-apex peak detection and an averaged peak smoothing width of 50–100 channels.

### 3.6. FAME GC-MS

FAMEs were prepared by transesterification of purified lipids by incubation for 20 h with 300−500 μL 3 N HCl in anhydrous methanol at 80 °C and extracted from the methanol solution using an equal volume of hexane. The hexane phase was dried under nitrogen, resuspended in a small amount (<100 μL) of 2:1 chloroform:methanol and analyzed on a Shimadzu QP2010-SE gas chromatograph with a 30-m SHRXI-5ms (5% phenyl) column with electron-impact mass spectrometry (EI-MS) peak detection and characterization. Shimadzu GC-MS post-run analysis software was used to analyze the mass spectrum of each chromatogram peak and automatically matched the spectra to a library of standard MS fragmentation patterns. In some cases, signal quality was sufficient to allow library identification with strong confidence (>95%). For many peaks, however, low signal strength supported only a qualitative assessment of acyl type (saturated, unsaturated, 2-OH or 3-OH methyl ester) based on the characteristic signature of each type of FAME in the EI-MS spectrum. To further identify these peaks, retention times were compared to those of commercial fatty acid standards run on the same instrument and column, including BAME (bacterial fatty acid methyl ester) standard mix, 37-component FAME standard mix, and C8-C22 FAME standard mix (all from Sigma-Aldrich, St. Louis, MO, USA).

## Supplementary Files

Supplementary File 1
