# Supplementary material for: Temperature-Dependence of Lipid A Acyl Structure in Psychrobacter cryohalolentis and Arctic Isolates of Colwellia hornerae and Colwellia piezophila
Source: Mar Drugs. 2015 Jul 30;13(8):4701–20. doi: 10.3390/md13084701 (PMC4557000; doi:10.3390/md13084701)
Supplement: Supplementary File 1 [file marinedrugs-13-04701-s001.docx]

**Supplementary Information**


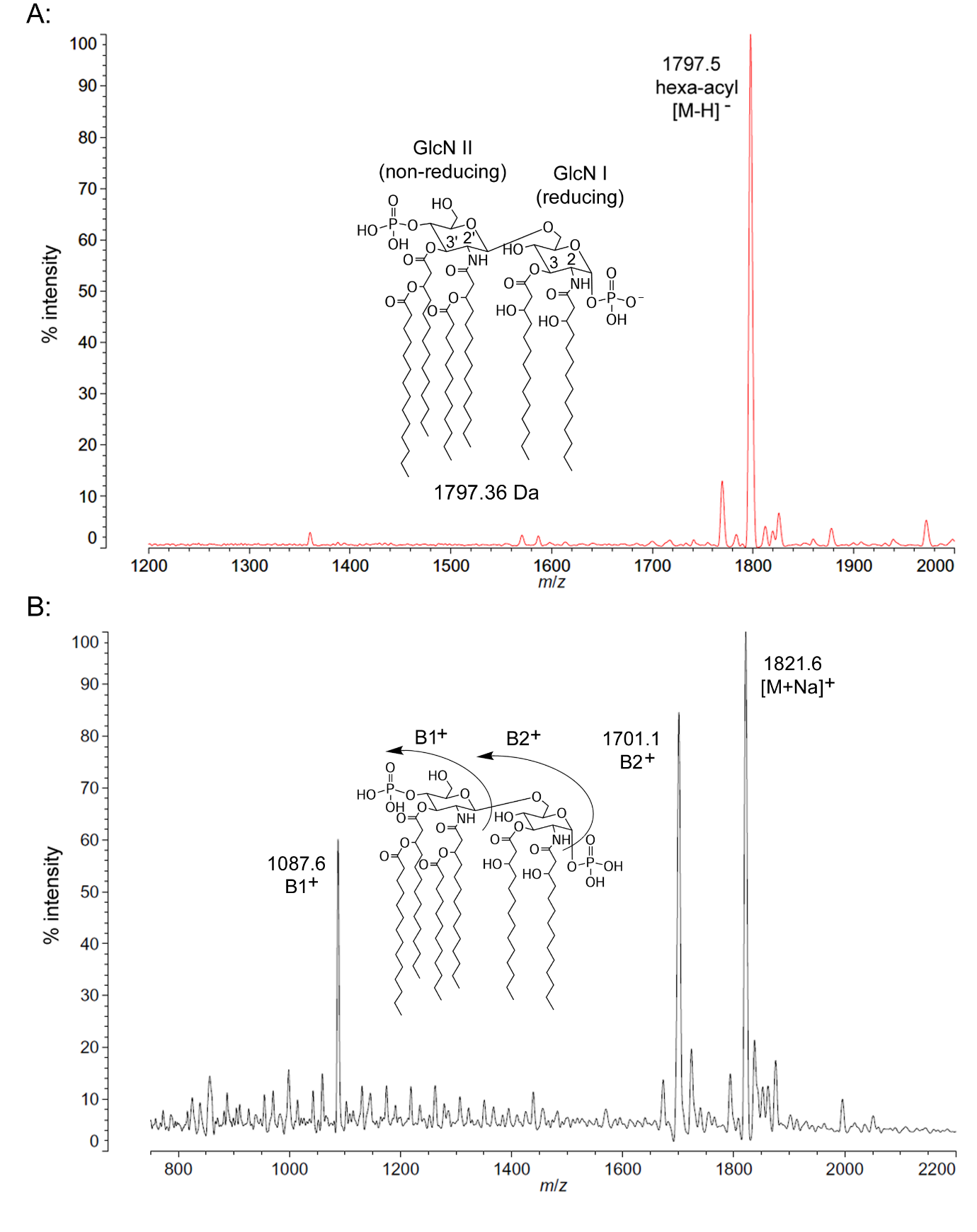


**Figure S1.** MALDI-TOF mass spectra of *E. coli* lipid A at 37 °C. Purified lipid A was prepared by mixing with ATT matrix as described in Materials and Methods*.* Spectral data were collected by Shimadzu Axima Confidence MS with a power setting of 70–80 and pulsed extraction at 2000 Da as the average of at least 1000 profiles in the negative linear mode (**A**) or the positive linear mode (**B**). Peak interpretation is shown on the spectra and
in Table 1.

**
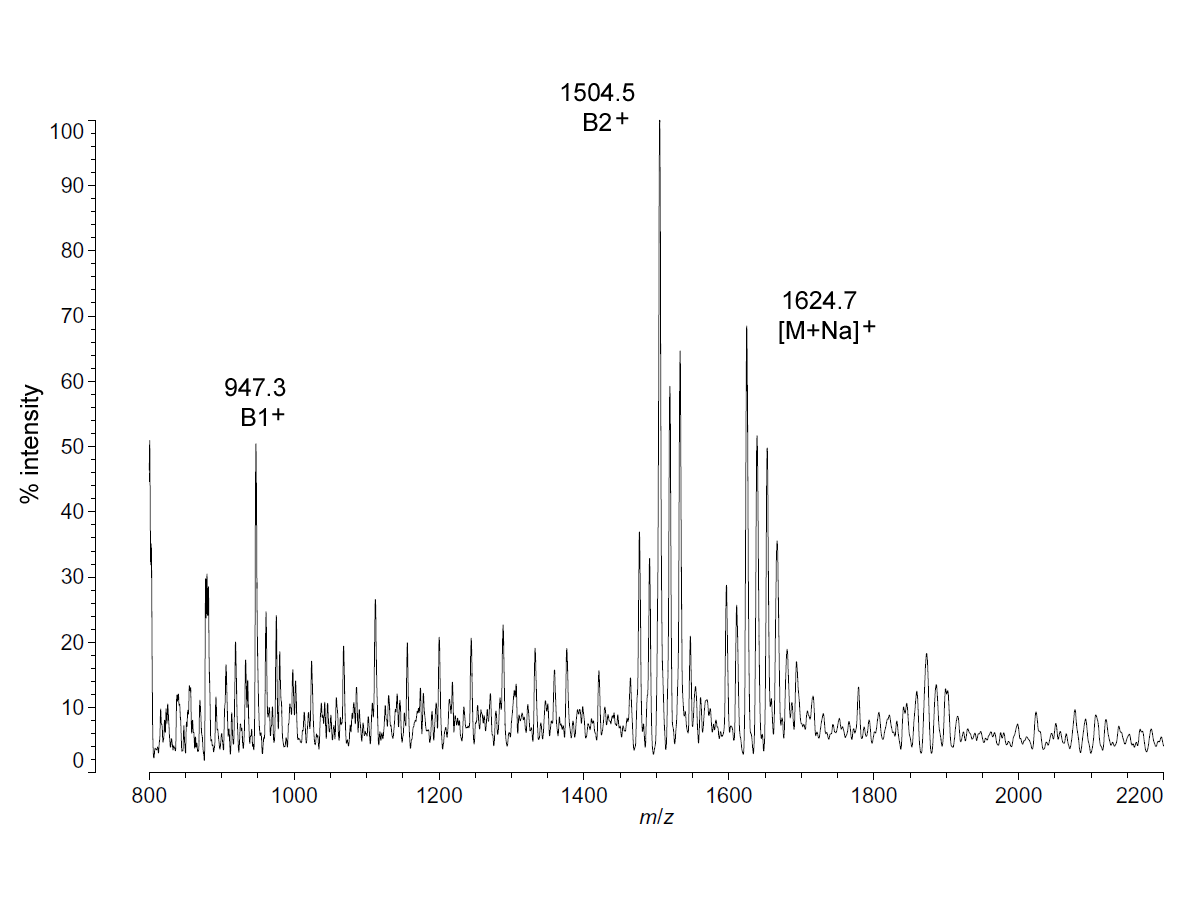
**

**Figure S2.** Positive ion MALDI-TOF mass spectra of *P. cryohalolentis* lipid A at 25 °C. Lipid A was prepared as described in Materials and Methods*.* Spectral data were collected by Shimadzu Axima Confidence MS with a power setting of 75 and pulsed extraction at 2000 profiles in the linear mode. Peak interpretation is shown on the spectra and in Table 1. The repetitive background peaks between 1000 and 1400 are due to the presence of sodium dodecyl sulfate aggregates derived from the purification method; background peaks below 950 are due to the MALDI-TOF ions of the matrix itself.

**Figure S3.** Negative ion MALDI-TOF mass spectra of *P. marina* lipid A at 4 °C and 15 °C. Lipid A was prepared as described in Materials and Methods*.* Spectral data were collected by Shimadzu Axima Confidence MS with a power setting of 75–80 and pulsed extraction at 2000 Da as the average of at least 700 profiles in the linear mode, displayed in 3D offset comparison by temperature (**A**), or as a stack of the main peak region (**B**). Peak interpretation and the structure of *P. marina* lipid A [1] are shown on the spectra.

**
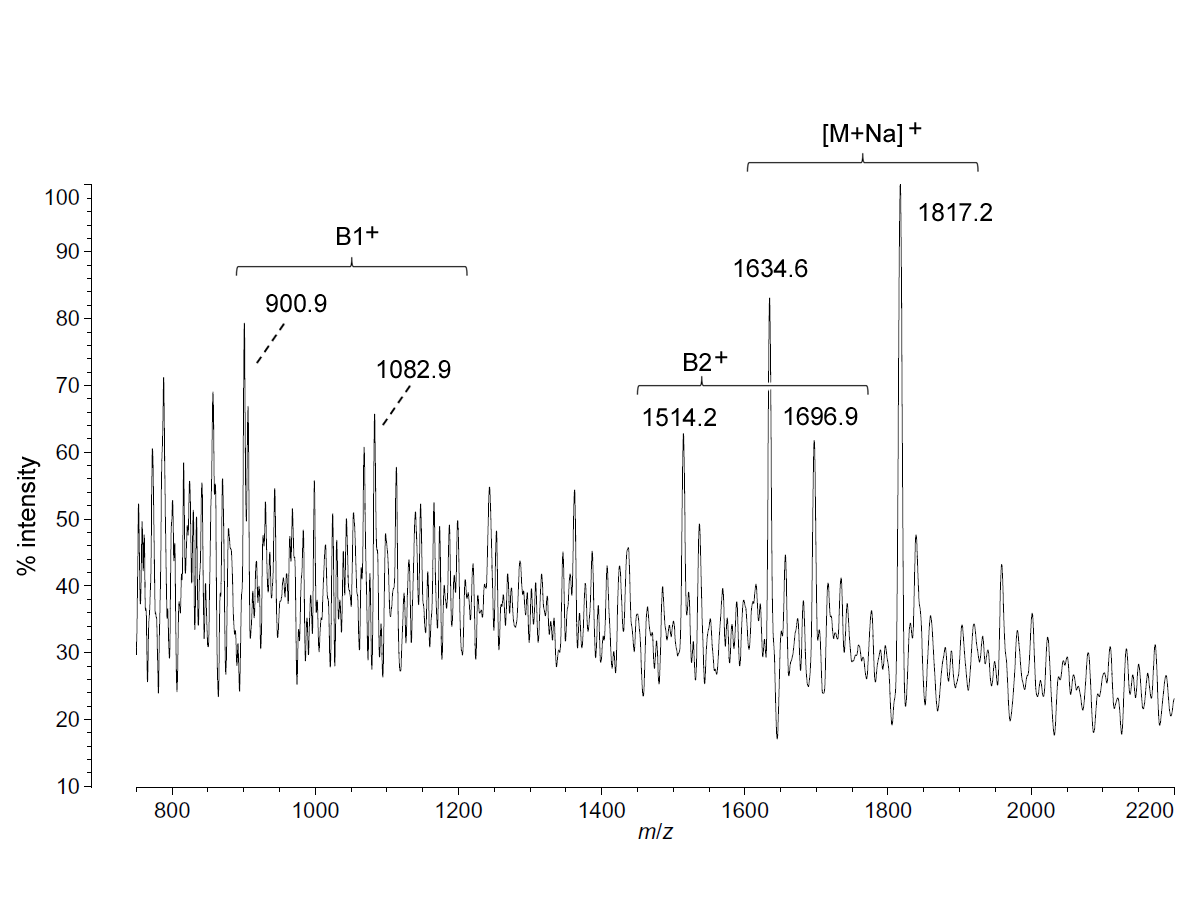
**

**Figure S4.** Positive ion MALDI-TOF mass spectra of *P. marina* lipid A at 4 °C. Lipid A was prepared as described in Materials and Methods*.* Spectral data were collected by Shimadzu Axima Confidence MS with a power setting of 90 and pulsed extraction at 2000 profiles in the linear mode. Peak interpretation is shown on the spectra.


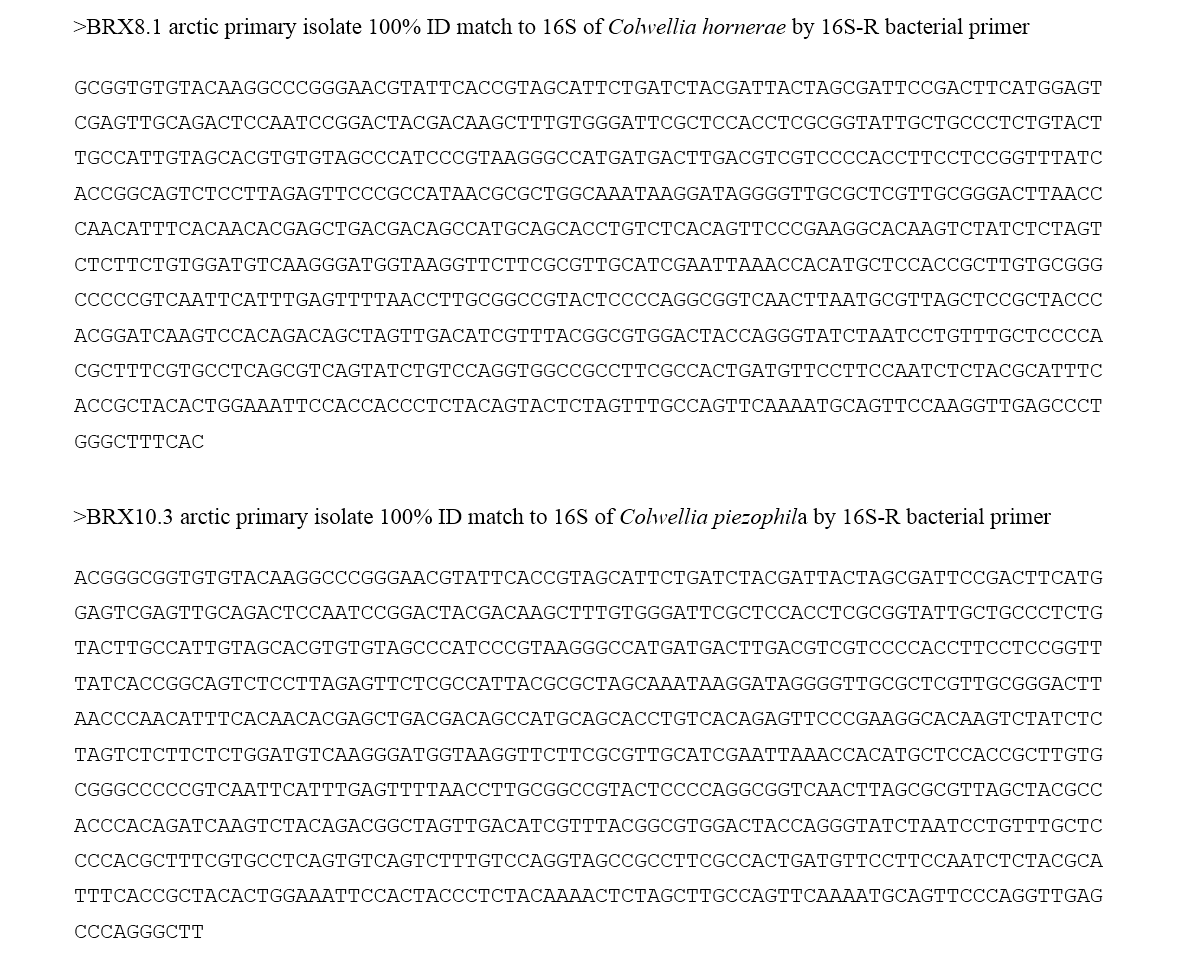


**Figure S5.** Sequence of 16S rDNA from *Colwellia hornerae* strain BRX8.1 and *Colwellia piezophila* strain BRX10.3. 16S rDNA was sequenced commercially by GENEWIZ
(South Plainfield, NJ, USA) by colony PCR and Sanger sequencing with 16S-reverse primer. The resulting sequence, shown here, was compared with the NCBI nr/nt curated database by BLAST algorithms (http://www.ncbi.nlm.nih.gov), establishing that BRX10.3 was unique among 19 Gram-negative isolates in that its 16S rDNA matches *C. piezophila* (100% identity) and not *C. hornerae*, as the rest of the isolates do. BRX8.1 is typical of the other Gram-negative isolates from the artic sea ice core, most of which (including BRX8.1) are a 100% match of
*C. hornerae*. 16S rDNA. These two sequences have been deposited with GenBank under accession numbers KR349200 and KR349201, respectively.

Reference

1. Sweet, C.R.; Alpuche, G.M.; Landis, C.A.; Sandman, B.C. Endotoxin structures in the psychrophiles *Psychromonas marina* and *Psychrobacter cryohalolentis* contain distinctive acyl features. *Mar. Drugs* **2014**, *12*, 4126–4147.

© 2015 by the authors; licensee MDPI, Basel, Switzerland. This article is an open access article distributed under the terms and conditions of the Creative Commons Attribution license (http://creativecommons.org/licenses/by/4.0/).
